# Jamming, fragility and pinning phenomena in superconducting vortex systems

**DOI:** 10.1038/s41598-020-68417-0

**Published:** 2020-07-15

**Authors:** Charles Reichhardt, Cynthia J. O. Reichhardt

**Affiliations:** Theoretical Division and Center for Nonlinear Studies, Los Alamos National Laboratory, Los Alamos, NM 87545 USA

**Keywords:** Superconducting properties and materials, Soft materials

## Abstract

We examine driven superconducting vortices interacting with quenched disorder under a sequence of perpendicular drive pulses. As a function of disorder strength, we find four types of behavior distinguished by the presence or absence of memory effects. The fragile and jammed states exhibit memory, while the elastic and pinning dominated regimes do not. In the fragile regime, the system organizes into a pinned state during the first pulse, flows during the second perpendicular pulse, and then returns to a pinned state during the third pulse which is parallel to the first pulse. This behavior is the hallmark of the fragility proposed for jamming in particulate matter. For stronger disorder, we observe a robust jamming state with memory where the system reaches a pinned or reduced flow state during the perpendicular drive pulse, similar to the shear jamming of granular systems. We show signatures of the different states in the spatial vortex configurations, and find that memory effects arise from coexisting elastic and pinned components of the vortex assembly. The sequential perpendicular driving protocol we propose for distinguishing fragile, jammed, and pinned phases should be general to the broader class of driven interacting particles in the presence of quenched disorder.

## Introduction

The jamming phenomenon refers to the cessation of flow in an assembly of interacting particles due to the formation of particle configurations which are stable against further motion even in the presence of a drive or load^1–4^. When the particle-particle interactions are short ranged, such as for grains or bubbles, there can be a well-defined specific density at which jamming occurs called point *J*^1,3–7^. When friction or additional constraints are introduced, such systems can jam at much lower densities under a drive and can exhibit two different regimes, the first of which is called a fragile phase where jamming occurs in the direction of shear but not in other directions^2,8,9^. In the second regime, driving can induce the formation of jammed phases such as shear jammed states^8,10,11^.

A phenomenon related to jamming is clogging, where the particle flow ceases due to a combination of particle-particle interactions and physical constraints such as a wall or posts, as observed in the flow of particles through a hopper^12,13^, bottlenecks^14^, channels^15,16^, and obstacle arrays^16–23^. A unifying theme in jamming and clogging is that particles must reach specific configurations in which the interactions create a network that can stop the flow. One consequence of this is that some jammed states can exhibit fragility, as introduced by Cates et al.^2^, where load-bearing force chains block the flow for the driving direction but not in other directions. The ideas of jamming and fragility have generally been applied to systems with short range or contact-only forces such as granular matter, emulsions and colloids; however, some of these ideas could be extended to systems with longer range interactions.

Another type of system that exhibits transitions from flowing to immobile states is assemblies of particles interacting with quenched disorder or pinning^24,25^. Examples of such systems include vortices in type-II superconductors^26,27^, electron crystals^28,29^, skyrmions^30,31^, emulusions^32^, colloids^33,34^, and active matter^35^. In these systems, the motion can be halted not only due to direct interactions between the particles and the pinning sites, but also by the blocking of unpinned particles through interactions with directly pinned particles. As a result, interactions play a significant role in the pinned state just as they do in the formation of jammed states. There are different types of depinning phenomena including elastic depinning, where the particles form a rigid solid with no exchanges or plastic events, and plastic depinning, where the particles can exchange places or tear past one another at and above depinning^24,25^. The transition from plastic flow to a pinned state should have the most similarities to a jamming transition from a mixing fluid state to a solid non-flowing state.

Despite the common features exhibited by both jamming and pinning, a connection between these two phenomena has not yet been established. One of the most studied systems that exhibits depinning is vortices in type-II superconductors, which can exhibit both plastic and elastic depinning transitions^24–27^. Driven vortices also exhibit many memory effects, in which the depinning threshold is strongly affected by the manner in which the pinned vortex configuration was created^36–43^. More recently it was shown that vortices can exhibit reversible to irreversible transitions or organize to specific dynamical states under periodic drives^44–47^, similar to the behavior found in colloidal systems and amorphous jammed systems^48–53^, suggesting that vortices can have properties resembling those in other systems that exhibit jamming or fragility.

**Figure 1:**
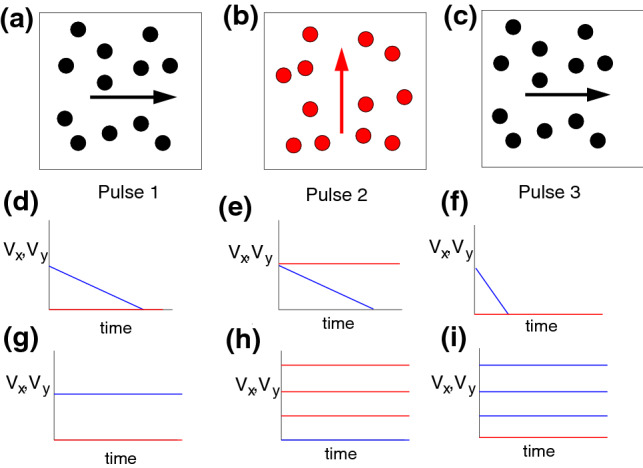
Schematic illustration of the driving protocol. (**a**) Pulse 1 ($p_1$) is applied along the *x* direction. (**b**) Pulse 2 ($p_2$) is applied along the *y* direction. (**c**) Pulse 3 ($p_3$) is applied along the *x* direction. (**d**) If the system organizes into a pinned state during pulse 1, the *x*-direction velocity $V_x$ (blue curve) decays to zero and the *y*-direction velocity $V_y$ (red curve) is always zero. (**e**) During pulse 2, $V_y$ has a fixed finite value and $V_x$ decays back to zero. (**f**) During pulse 3, $V_y$ is zero and $V_x$ exhibits a transient signal before the entire system reaches a pinned state. (**g**) An alternative response showing a finite steady state value of $V_x$ during pulse 1. (**h**) The response in panel (**g**) is followed during pulse 2 by a finite $V_y$ value that may be larger than, equal to, or smaller than the value of $V_x$ during pulse 1, while $V_x$ during pulse 2 is zero. (**i**), The subsequent response in pulse 3 has a $V_x$ value that may be larger than, equal to, or smaller than the value of $V_x$ during pulse 1, while $V_y$ is zero. If the system has no memory, the response in the driving direction will be the same during all three pulses.

An open question is how to distinguish between jamming and pinning-dominated behaviors in superconducting vortices or in general systems with quenched disorder. Here we propose a method to use a pulsed drive protocol to characterize these different phases for vortices interacting with random disorder. We first apply a drive pulse to the system in one direction for a fixed period of time, then shut off that drive and apply a second drive pulse in the perpendicular direction for the same duration of time. We then shut off the second pulse and apply a third pulse in the original direction to test whether the system retained any memory of the first driving pulse. In Fig. 1a,b,c we illustrate a schematic of the driving protocol. During pulse 1, termed $p_1$, the drive is applied in the *x* direction. The second driving pulse $p_2$ is applied along the *y* direction. Finally, pulse $p_3$ is applied along the *x* direction. If the system has no memory, the response in the driving direction is identical during all three pulses.

We find that when the number of vortices is greater than the number of pinning sites, two different types of jamming behaviors appear. For weak disorder, the system exhibits fragility and the vortices organize into a clogged configuration during $p_1$, as shown schematically in Fig. 1d. This fragile configuration is unstable to a change in the driving direction, and thus during $p_2$ the vortices are unable to form a new network that can block the flow in the *y* direction, resulting in a finite value of $V_y$ as illustrated in Fig. 1e. During $p_3$ when the drive is reapplied in the original *x* direction, the system retains a memory of the initially pinned state, as indicated in Fig. 1f. When the drive is larger, the vortices may flow during all drive pulses, as shown in Fig. 1g–i, where the flow in the driving direction has a constant value in the absence of memory but varies with driving direction when memory is present. For stronger disorder, we observe a more robust jamming behavior similar to that found in shear jammed systems. Here, even if $p_1$ produces steady state flow, the application of $p_2$ lowers the mobility of the flow, while under repeated perpendicular switching of the driving direction, the system eventually reaches a fully jammed state. In the pinning-dominated regime, if the system reaches a pinned state during $p_1$, then it remains pinned during $p_2$ and all subsequent drive pulses. For weak pinning or low pinning density, we find that the memory disappears and the response is more elastic, with rearrangements occurring among the directly pinned vortices.

We map the regions in which fragile and pinned phases occur as a function of drive strength and pinning density. We also discuss how these results could be generalized to other systems that contain quenched disorder or that exhibit nonequilibrium memory effects. Our results indicate that different types of memory effects can be used to distinguish fragile jammed states from robustly jammed states. In addition, the ability of the system to retain a memory of different driving directions could be exploited to create new types of computing devices such as a memristor^54,55^.

## Simulation and system

We consider a two-dimensional $L \times L$ system of rigid vortices with periodic boundary conditions in the *x* and *y*-directions. The system contains $N_{v}$ vortices and $N_{p}$ pinning sites, with vortex density $n_v=N_v/L^2$, pinning density $n_p=N_p/L^2$, and a vortex to pin ratio of $R=n_v/n_p$. The vortex dynamics is obtained using the following overdamped equation of motion:

1 $$\begin{aligned} \alpha _d {\mathbf{v}}_{i} = {\mathbf{F}}^{vv}_{i} + {\mathbf{F}}^{p}_{i} + {\mathbf{F}}^{D} . \end{aligned}$$

The vortex velocity is $\mathbf{v}_{i} = {d \mathbf{r}_{i}}/{dt}$ and $\alpha _d$ is the damping constant which we take equal to 1. The vortex-vortex interactions are given by $\mathbf{F}^{vv}_{i} = \sum ^{N}_{j=1}F_{0}K_{1}(r_{ij}/\lambda ){\hat{\mathbf{r}}_{ij}}$, where $F_{0} = \phi ^2_{0}/2\pi \mu _{0}\lambda ^3$, $\phi _{0}$ is the elementary flux quantum, $\lambda$ is the London penetration depth, and $\mu _{0}$ is the permittivity. The distance between vortex *i* and vortex *j* is $r_{ij} = |\mathbf{r}_{i} - \mathbf{r}_{j}|$ and $K_{1}$ is the modified Bessel function, which falls off exponentially for large $r_{ij}$. For $r_{ij}/\lambda > 6.0$ the interaction between vortices is weak enough that it can be cut off for computational efficiency, and larger cutoffs produce negligible differences in the results for the range of vortex densities we consider. In the absence of pinning, the vortices form a triangular lattice with lattice constant *a*. The $N_{p}$ pinning sites are placed in random but non-overlapping positions. Each pinning site is modeled as a parabolic trap of range $r_{p}$ that can exert a maximum pinning force of $F_{p}$ on a vortex. The vortex-pin interaction has the form $\mathbf{F}_i^{p} = \sum _j^{N_p}(F_{p}/r_{p})(r_{i} -r_{j}^{(p)})\Theta (r_{p} -|\mathbf{r}_{i} - \mathbf{r}_{j}^{p}|)$. Our unit of length is $\lambda$ and we set $L = 36$, $F_p=1.0$, and $r_{p} = 0.25$. We focus on the case of a vortex density of $n_{v}= 1.67$ with $N_v=2160$ vortices and vary the pin density from $n_p=0$ to $n_p=2.65.$

The initial vortex configurations are obtained by starting the system at a high temperature and gradually cooling to $T = 0.0$. After annealing, we apply a series of constant drives to the vortices, $\mathbf{F}_D=F_D{\hat{\alpha }}$, where $\hat{\alpha }=\hat{\mathbf{x}}$ during drive pulses $p_1$ and $p_3$ and $\hat{\alpha }=\hat{\mathbf{y}}$ during drive pulse $p_2$. We measure the average velocity per vortex in the *x* and *y* directions, $\langle V_{x}\rangle = N_v^{-1}\sum ^{N_v}_{i=1} \mathbf{v}_{i}\cdot \hat{\mathbf{x}}$ and $\langle V_{y}\rangle = N_v^{-1}\sum ^{N_v}_{i=1} \mathbf{v}_{i}\cdot \hat{\mathbf{y}}$. Drive pulse $p_1$ is applied along the *x*-direction for a duration of $\tau = 2.2\times 10^6$ simulation time steps. During drive pulse $p_2$, we shut off the *x* direction driving and apply a drive of the same magnitude $F_D$ along the *y* direction for the same duration $\tau$. If the system has no memory of the initial drive, then the average velocity *in the driving direction* will be equal during both pulses, $|\langle V_x\rangle ^{1}| = |\langle V_y\rangle ^{2}|$. After the first two pulses, we can continue the pulse sequence by applying a third pulse $p_3$ in the same direction as $p_1$ to test for additional memory effects.

## Results

**Figure 2:**
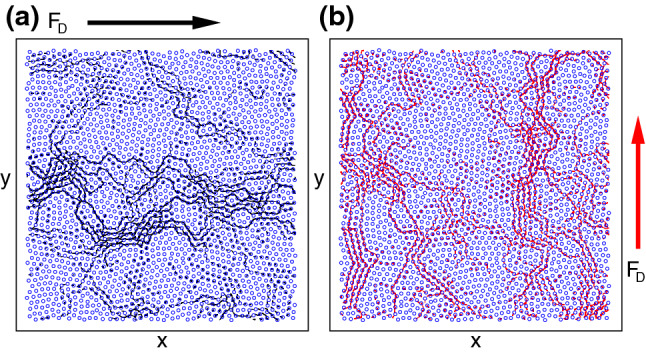
Fragile phase flow. Vortex positions (circles) and trajectories (lines) in a system with $n_{p} = 1.0$. The pinning site locations are not shown. We apply a drive pulse $p_1$ of magnitude $F_D=0.045$ along the *x* direction (black arrow) for a duration of $\tau$ simulation time steps, followed by a drive pulse $p_2$ of the same magnitude and duration along the *y* direction (red arrow). (**a**) Motion during $p_1$, where there is a combination of pinned and flowing vortices. (**b**) Motion during $p_2$, where the amount of flow is increased and the flow direction has rotated to follow the drive.

In Fig. 2 we plot the vortex locations and trajectories over a fixed time period for a system with $F_{p}= 1.0$ and $n_{p} = 1.0$ under driving of magnitude $F_D=0.045$. During pulse $p_1$ in Fig. 2a, there is a combination of pinned and flowing vortices, and the motion is mostly along the *x* direction, parallel to the drive. During pulse $p_2$, which is applied along the *y*-direction, Fig. 2b illustrates that there is now a greater amount of motion in the system. This indicates that when the drive is applied initially in the *x*-direction, the flow organizes to a partially jammed state in which a portion of the flow is blocked in the *x* direction but not in the *y* direction. During pulse $p_3$, the flow is identical to that shown in Fig. 2a. If $F_{D}$ is reduced, we find states in which the system organizes to a pinned configuration during $p_1$ but is still able to flow when the drive is switched to the *y* direction during $p_2$. For very low drives of $F_{D} < 0.03$, the system becomes pinned for both driving directions.

**Figure 3:**
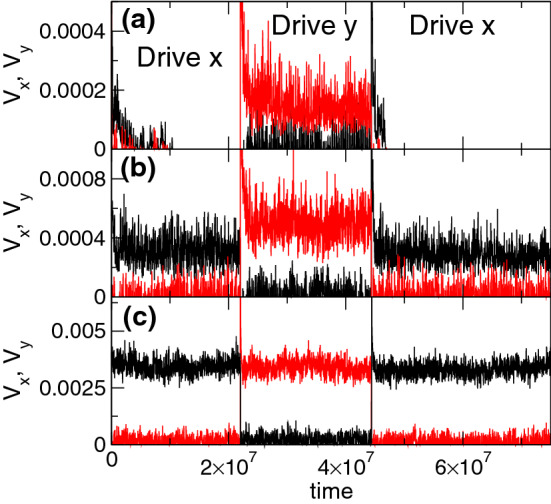
Fragile phase velocity response. The time series of the velocities $V_{x}$ (black) and $V_{y}$ (red) for the system in Fig. 2 with $n_{p} = 1.0$ for pulse $p_1$ applied along the *x* direction, pulse $p_2$ applied along the *y* direction, and pulse $p_3$ applied along the *x* direction within the fragile phase. (**a**) At $F_{D} = 0.035$, the system organizes to a jammed state during $p_1$, flows during $p_2$, and returns rapidly to a pinned state during $p_3$. (**b**) At $F_{D} = 0.045$, the system organizes to a flowing state in $p_1$, exhibits a state with higher flow during $p_2$, and returns to the first flow level during $p_3$. (**c**) At $F_{D} = 0.08$, the memory effect is lost and the velocity in the driving direction is the same during all drive pulses.

To focus more specifically on the memory effects, in Fig. 3a we plot the time series of $V_{x}$ and $V_{y}$ for the system in Fig. 2 at $F_{D} = 0.035$. During pulse $p_1$, $V_{y} = 0$ while $V_{x}$ initially has a finite value and gradually drops to zero as the system organizes into a pinned state. When pulse $p_2$ is applied, the *x* direction motion remains absent with $V_{x} = 0$, but $V_{y}$ is finite and does not decay to zero. This indicates that the system retains a memory of the initial driving direction, and that the jammed state which forms for *x* direction driving is unstable against driving in a different direction, which is a key signature of fragility^2^. During pulse $p_3$, when the drive is applied in the *x*-direction again, the system returns to a pinned state with $V_{x}=V_y=0$, indicating that a memory of the initial driving in *x* has persisted. We note that if we continue to repeat alternating driving pulses in the *x* and *y* directions, there is always flow for *y* direction driving and no flow for *x* direction driving. The same system under a drive of magnitude $F_D=0.045$ is illustrated in Fig. 3b. Here $V_{x}$ remains finite during pulse $p_1$ and $V_{y}$ is also finite during pulse $p_2$, but the flow in the direction of drive is larger for the *y* direction driving than for the *x* direction driving. In this case, during pulse $p_1$, the system organizes to a state in which only a portion of the flow is jammed in the *x* direction while a smaller portion is jammed in the *y* direction. This occurs because many of the pinned vortices from $p_1$ remain pinned during $p_2$, as shown in Fig. 2a, so that even though there is flow for both driving directions, there is still a memory effect due to these pinned vortices. During pulse $p_3$, $V_x$ returns to the same value it had in $p_1$, so the system remembers the flow state that formed during the original driving pulse. Figure 3c shows that in the same system at a higher drive of $F_{D} = 0.08$, $V_{x}$ in pulses $p_1$ and $p_3$ has the same value as $V_{y}$ in pulse $p_2$. Here, many of the vortices are unpinned much of the time, causing the destruction of the memory effect, which is replaced by a uniform response that is insensitive to the driving direction. When $F_{D} < 0.0325$, the system reaches a pinned state for both directions of driving; however, the transient time required to reach the jammed state during pulse $p_2$ in the *y* direction is considerably longer than that for the initial pulse $p_1$ in the *x* direction.

**Figure 4:**
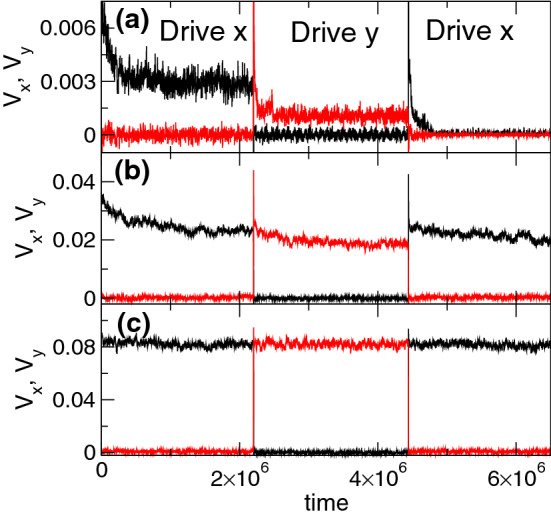
Jammed phase velocity response. The time series of the velocities $V_{x}$ (black) and $V_{y}$ (red) for a system with $n_p=1.5$ where jamming effects appear. Driving pulse $p_1$ is applied along the *x* direction, pulse $p_2$ is applied along the *y* direction, and pulse $p_3$ is applied along the *x* direction. (**a**) At $F_{D} = 0.3$, the system organizes to a flowing state during $p_1$, exhibits reduced flow during $p_2$, and has almost no flow during $p_3$. (**b**) At $F_{D} = 0.475$, during $p_1$ the system organizes to a flowing state, followed by a state with lower flow during $p_2$ and a return to the higher flow level during $p_3$. (**c**) At $F_{D} = 0.6$, $V_{x}$ for *x* direction driving is equal to $V_{y}$ for *y* direction driving, and the pulse memory effect is lost.

The memory effect indicative of fragility, where the response in the driving direction is larger during $p_2$ than during $p_1$, occurs over the range $0.45< n_p < 1.25$ when $n_v = 1.67$. For $1.25 \le n_{p} < 2.25$, we observe a reversal of the memory effect in which the system exhibits a more robust jamming effect similar to that found in shear jammed systems. In Fig. 4a we plot $V_{x}$ and $V_{y}$ as a function of time for a system with $n_{p} = 1.5$ at $F_{D} = 0.3$. During pulse $p_1$, $V_{x}$ decreases before reaching a finite steady state value. When pulse $p_2$ is applied, $V_{y}$ is lower than the steady state value of $V_x$ during pulse $p_1$, indicating that rotation of the drive by 90$^\circ$ has allowed the system to access a configuration with a higher amount of jamming. During pulse $p_3$, $V_{x}$ drops to nearly zero, indicating a further reorganization. For lower $F_{D}$, the system can reach a pinned state during pulse $p_2$ and is completely pinned in pulse $p_3$. In Fig. 4b, for the same system at $F_{D} = 0.475$, $V_y$ during pulse $p_2$ is lower than $V_x$ during pulse $p_1$, but during pulse $p_3$, $V_x$ actually jumps up to a higher value since almost all of the possible jammed configurations have already been accessed by the system. Figure 3c shows that at $F_{D} = 0.6$, the response in the driving direction is the same during all drive pulses and the memory effect is lost.

**Figure 5:**
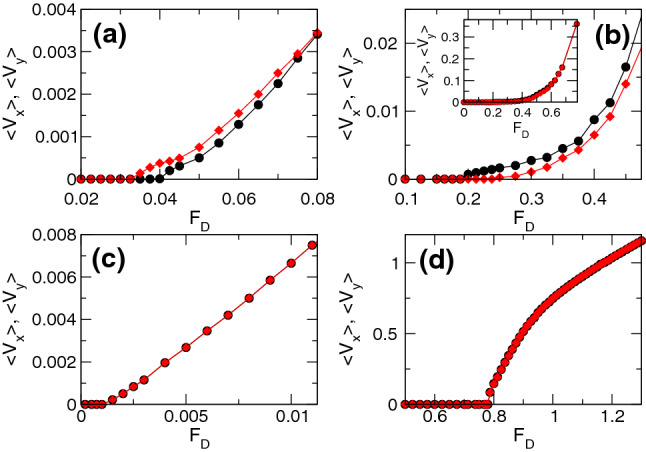
Average velocity response under varied pulse magnitude. $\langle V_{x}\rangle$ at the end of pulse $p_1$ (black circles) and $\langle V_{y}\rangle$ at the end of pulse $p_2$ (red diamonds) vs $F_{D}$. (**a**) The fragile phase for the system in Figs. 2 and 3 with $n_{p} = 1.0$, where $\langle V_{y}\rangle$ in $p_2$ is higher than $\langle V_{x}\rangle$ in $p_1$. (**b**) The jammed phase for the system in Fig. 4 with $n_{p} = 1.5$ where $\langle V_{y}\rangle$ in $p_2$ is lower than $\langle V_{x}\rangle$ in $p_1$. The inset shows the same data out to higher values of $F_D$. (**c**) The elastic phase for a system with $n_{p} = 0.1$, where the response in the driving direction is the same in both $p_1$ and $p_2$ and there is no memory effect. (**d**) The pinning dominated phase at $n_{p} = 2.625$, where there is no memory effect.

We can characterize the different phases by measuring the average velocity $\langle V_x\rangle$ at the end of pulse $p_1$ and $\langle V_{y}\rangle$ at the end of pulse $p_2$ for varied $F_{D}$. In Fig. 5a we plot $\langle V_{x}\rangle$ and $\langle V_{y}\rangle$ versus $F_{D}$ for the system in Figs. 2 and 3 with $n_p=1.0$ which is in the fragile phase. Here, the system reaches a pinned state during both $p_1$ and $p_2$ when $F_{D} \le 0.0325$, while for $0.0325< F_{D} < 0.04$, the system jams during $p_1$ but flows during $p_2$. For $0.04 \le F_{D} < 0.08$, both $\langle V_x\rangle$ and $\langle V_y\rangle$ are finite, but the response during $p_2$ is larger than that during $p_1$. The ratio $\langle V_{x}\rangle /\langle V_{y}\rangle$ for pulses $p_1$ and $p_2$ gradually approaches $\langle V_{x}\rangle /\langle V_{y}\rangle =1.0$ as $F_{D}$ increases, until for $F_{D} \ge 0.08$, the response is the same for both driving directions and the memory effect is lost. In Fig. 5b, we plot $\langle V_x\rangle$ and $\langle V_y\rangle$ versus $F_D$ for the system in Fig. 4 with $n_{p} = 1.5$, where jamming behavior appears. For $0.1875< F_{D} < 0.25$, the system flows in the *x* direction during pulse $p_1$, and is pinned in the *y* direction during $p_2$. When $0.25 \le F_{D} < 0.6$, there is a finite response during both $p_1$ and $p_2$, but the response is smaller in $p_2$. For $F_{D} \ge 0.6$, the response becomes isotropic as shown in the inset of Fig. 5b. For samples with $n_{p} < 0.4$, the response becomes mostly elastic and the memory effects disappear, as shown in Fig. 5c where we plot $\langle V_{x}\rangle$ and $\langle V_{y}\rangle$ versus $F_{D}$ for a system with $n_{p} = 0.1$. The system is pinned for $F_{D} < 0.001$, but the response is isotropic for higher $F_{D}$. Samples with $n_{p} > 2.25$ are in the pinning-dominated regime, where there is again no memory effect, as shown in the plot of $\langle V_{x}\rangle$ and $\langle V_{y}\rangle$ in Fig. 5d for a system with $n_{p} = 2.625$. In the pinning-dominated regime, almost every vortex can be pinned directly at a pinning site, so the collective effects associated with jamming or fragility are lost.

**Figure 6:**
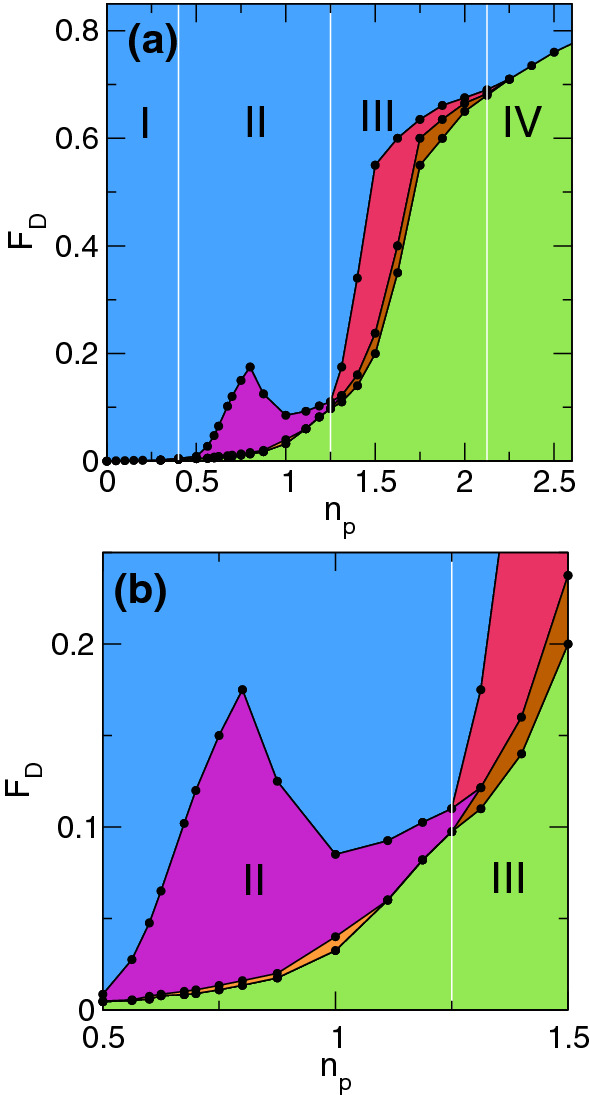
Dynamic phase diagram as a function of $F_D$ **vs** $n_p$ obtained from a series of velocity versus pulse force measurements. (**a**) Green: pinned phase where the system reaches a pinned state during both $p_1$ and $p_2$. Blue: isotropic flow phase where the response in the driving direction has the same value for both $p_1$ and $p_2$. Orange: the fragile phase in which the system is pinned during $p_1$ and flowing during $p_2$. Purple: the fragile phase in which the system is flowing in $p_1$ and has a larger magnitude of flow during $p_2$. Brown: the jammed phase where the system flows in $p_1$ and is pinned in $p_2$. Red: the jammed phase in which there is flow in $p_1$ and reduced flow in $p_2$. The white lines indicate the divisions between the four phases that appear as a function of pinning density below the isotropic flow regime. Phase I: elastic regime with no memory. Phase II: fragile jammed state with memory. Phase III: robust jammed state with memory. Phase IV: pinned state with no memory. (**b**) A blow up of panel (**a**) in the region of the fragile to jammed crossover.

By conducting a series of simulations for varied pulse magnitude $F_{D}$ and pinning density $n_{p}$, we can construct a dynamic phase diagram highlighting the four different phases as a function of $F_D$ versus $n_p$, as plotted in Fig. 6a. In the pinned phase, the system reaches a pinned state during both $p_1$ and $p_2$. For the isotropic flow state, the velocity response in the driving direction is the same during both $p_1$ and $p_2$. The fragile response states include the regime in which the system is pinned during $p_1$ and flowing during $p_2$, as well as the parameter range in which the system is flowing during $p_1$ and flowing faster during $p_2$. The jammed phases consist of states in which the system is flowing during $p_1$ but pinned during $p_2$ as well as the regime in which there is flow during $p_1$ and reduced flow during $p_2$. In Fig. 6b, we show a blowup of Fig. 6a near the fragile to jamming crossover region to illustrate more clearly the different fragile and jammed phases. The vertical white lines in Fig. 6a indicate the four phases as a function of pinning density. These are the elastic regime with no memory, marked as Phase I, which appears for $n_{p} < 0.4$, the fragile jammed state with memory, marked as Phase II, for $0.4 \le n_{p} < 1.25$, the robust jamming state with memory, marked as Phase III, for $1.25 \le n_{p} < 2.125$, and the pinned state with no memory, marked as Phase IV, for $n_{p} \ge 2.125$. Near the boundary between fragile and jammed behavior, multiple reversals in the velocity force curves can occur such that the system may show a jamming behavior at lower drives but a fragile behavior at higher drives. At sufficiently high drives, in the blue region, the response becomes isotropic. We have constructed a similar phase diagram for a vortex density of $n_{v} = 0.75$ as a function of $F_D$ versus $n_{p}$ and find phase boundaries similar to those shown in Fig. 6, indicating the robustness of the results. In general, pinning dominated phases occur when $n_{v}/n_{p} < 1.25$, where each vortex can be trapped directly by a pinning site so that the vortex-vortex interaction energies become unimportant.

**Figure 7:**
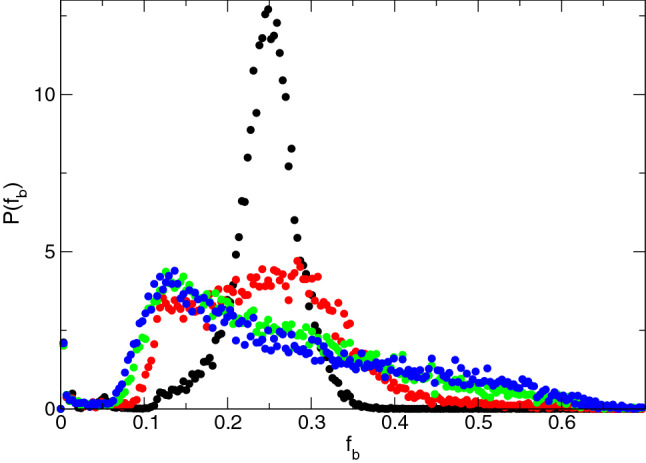
Nearest neighbor forces in four phases. Distribution $P(f_b)$ of nearest-neighbor vortex-vortex interaction forces, histogrammed using five different realizations. In the elastic phase I at $n_p = 0.1$ (black), there is a single narrow peak in $P(f_b)$. In the fragile phase II at $n_p=0.7$ (red), there are two peaks on either side of a plateau, and in both the jammed phase III at $n_p=1.5$ (green) and the pinned phase IV at $n_p=2.5$ (blue) the distribution has a broad tail.

**Figure 8:**
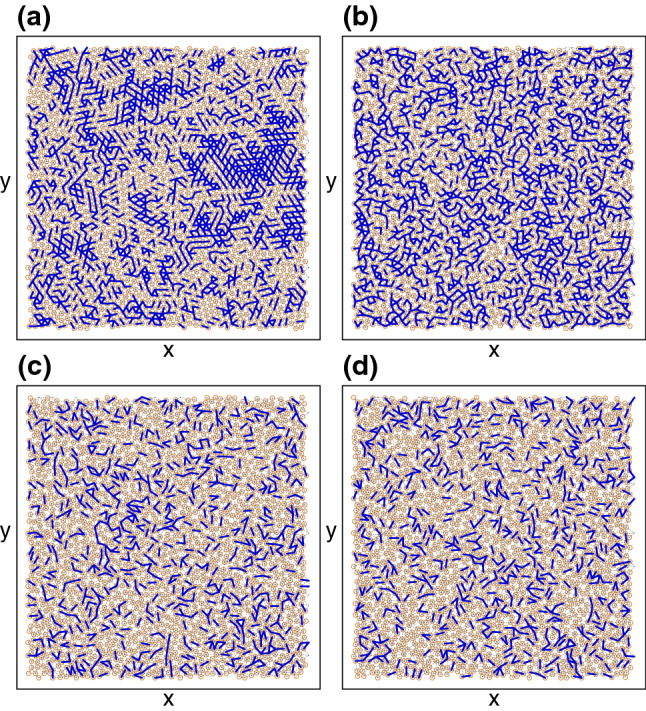
Bond networks. Images of the network of nearest-neighbor bonds between vortices for example realizations of each system plotted in Fig. 7, showing the vortex positions (orange circles), bonds with a probability of observation $P(f_b) \ge 0.9P_{\mathrm{max}}$ (heavy blue lines), and the remaining bonds (thin dashed black lines), where $P_{\mathrm{max}}$ is the maximum value of $P(f_b)$. (**a**) The elastic phase I at $n_p=0.1$, where there are many bonds with nearly the same strength since the crystalline structure of the vortex lattice prevents the vortices from adapting to the pinning landscape. (**b**) The fragile phase II at $n_p=0.7$, where there is a broad plateau in $P(f_b)$ that produces a highly adaptive bond network that can retain a memory of the driving direction. (**c**) The jammed phase III at $n_p=1.5$. (**d**) The pinned phase IV at $n_p=2.5$.

To gain insight into the onset of the different phases, we measure the distribution of the forces experienced by the vortices. We first identify the true nearest neighbors of each vortex using a Voronoi construction, and assign each nearest neighbor pair to a bond. Using the length of each bond, we compute the interaction force $f_b$ between each pair of neighboring vortices, and histogram this quantity using five different realizations for Phases I through IV. In Fig. 7 we plot the distribution function $P(f_b)$ for the elastic phase I at $n_{p} = 0.1$, the fragile phase II at $n_{p} = 0.7$, the jammed phase III at $n_{p} = 1.5$, and the pinned phase IV at $n_{p} = 2.5$. For the elastic phase we find a single relatively narrow peak since most of the vortices have six neighbors and the vortex-vortex spacing is fairly uniform. In Fig. 8a we show an image of the vortex-vortex nearest neighbor bond network. Highlighted in bold are those bonds whose frequency of observation $P(f_b)$ is within 10% of $P_{\mathrm{max}}$, $P(f_b) \ge 0.9 P_{\mathrm{max}}$, where $P_{\mathrm{max}}$ is the maximum value of $P(f_b)$. In the elastic regime, there are large regions of sixfold ordering, producing large patches of bonds with nearly the same value of $f_b$. Since the vortex-vortex interactions dominate in this regime, few rearrangements of the nearest neighbor structure occur above the depinning transition during drive pulse $p_1$, and the bond network structure remains unchanged during drive pulse $p_2$. As a result, the system has no memory of the driving direction. In the fragile phase II, $P(f_b)$ has two peaks on either side of a broad plateau, as shown in Fig. 7 for a sample with $n_p=0.7$. The peak at higher $f_b$ is associated with the remaining elastic component of the vortex lattice, while the peak at lower $f_b$ arises due to increasing plastic distortions of the lattice by the underlying pinning. Due to the broad plateau, a wide range of values of $f_b$ meet the criterion $P(f_b)\ge 0.9 P_{\mathrm{max}}$, producing a force network of the type illustrated in Fig. 8b. The large elastic patches that appeared in the elastic phase are replaced by small clusters of bonds that have local orientation but no global orientation. These disordered regions can undergo strong rearrangements in order to create a percolating force network that is able to stop the flow in the *x* direction during drive pulse $p_1$. A portion of these bonds break and rearrange during drive pulse $p_2$, but another portion of the bonds are preserved, so that when drive pulse $p_3$ is applied, enough of the network that appeared in $p_1$ is still present that the system is able to reform a similar network and maintain a memory of its original driving direction. In the jammed phase III, as shown in Fig. 7 for $n_p=1.5$, the elastic peak in $P(f_b)$ disappears and we instead find a peak at lower $f_b$ along with a broad tail extending out to high values of $f_b$. Here the pinning interaction energy is beginning to overwhelm the vortex-vortex interaction energy, producing some long neighbor bonds with forces lower than those found in an elastic lattice along with other short neighbor bonds with forces higher than those that would be present in an elastic lattice. Due to the highly skewed shape of $P(f_b)$, there are fewer bonds with $P(f_b)\ge 0.9 P_{\mathrm{max}}$, and these bonds form a scattered and non-percolating structure, as illustrated in Fig. 8c. Similar behavior of both $P(f_b)$ and the bond network structure appears in the pinned phase IV, as shown in Figs. 7 and 8d for samples with $n_p=2.5$. In the jammed phase, during successive drive pulses the system gradually approaches a state resembling the pinned phase, where all motion has ceased and all memory has disappeared. The force distributions shown in Fig. 7 have distinctive signatures depending on whether the system is elastic, fragile, or jammed/pinned. As a result, it may be possible to determine whether elastic, fragile, jammed or pinned behavior is likely to occur by examining the vortex configurations even before a drive is applied. This can be achieved experimentally using a variety of imaging techniques^56–59^.

## Discussion

Our results indicate that in experiments, fragile or jamming behaviors would be most relevant for regimes in which the number of vortices is greater than the number of pinning sites or in samples where the pinning is strongly inhomogeneous. Strong memory effects or metastable effects are generally observed in superconductors that are either very clean or have only weak pinning, which is consistent with our findings. Jamming and fragility effects should be absent in or near the elastic depinning regime, where the vortex lattice is almost completely ordered, as well as in systems with strong pinning. Our results should be general to the broader class of particle-like systems interacting with pinning, including magnetic skyrmions, Wigner crystals, and colloids with random pinning. It is likely that similar effects would arise in systems where the particle-particle interactions are short ranged or have a hard sphere character, and this will be explored in a future work. The general protocol of applying a sequence of perpendicular drives could also be used in systems without quenched disorder, where it could provide a new method for studying random organization processes of the type found in periodic shearing of dilute colloidal systems. For instance, this protocol could be applied to dense amorphous soft solids to see if such systems also exhibit a fragile to jamming crossover. It is also possible that different pulse protocols could be used to determine whether the system retains different types of memory, similar to what has been observed in periodically sheared systems^60^.

In experiments, it would be possible to use a crossed driving geometry that permits the application of mutually perpendicular driving currents. There are also a variety of techniques that can be used to add a well defined number of pinning sites to the system in random or periodic arrangements^61–63^, permitting the ratio of the number of vortices to the number of pinning sites to be tuned by varying the applied magnetic field. It would also be interesting to test whether periodic pinning arrays produce extended regions of fragile states or reduce the occurrence of fragile states. The conversion from simulation parameters to physical parameters depends upon the choice of material; however, as an example we consider the system studied by Dobroka et al.^46,64^, where memory effects for driving in one direction have already been observed. In this case, the ac pulse frequency is in the Hz to kHz regime; however, longer or shorter pulse durations would be adequate for the measurement if the system reaches a steady dynamical state. For vortices that are displaced on the order of 0 to 20$\mu$m during a single drive pulse, typical voltages would be in the range of 10 mV, which are readily experimentally accessible.

## Summary

We have proposed a protocol involving a sequence of mutually perpendicular driving forces for determining whether a system with quenched disorder is in an elastic, pinned, jammed, or fragile state. In the fragile state, the system is able to organize to a pinned state during the first drive pulse, flows during the second drive pulse which is applied perpendicular to the first pulse, but then returns to the initial pinned configuration during the third pulse, where the drive is applied in the original direction. The fragile state is associated with a particle configuration that is pinned only in the direction of drive but not in other directions. In the jammed phase, the system can reach a steady state flow during the first drive pulse, but the application of the second pulse can cause a reduction in the mobility, while during the third pulse the system can cease flowing and reach a jammed state similar to the shear jamming behavior found in frictional granular materials. For weak disorder, the memory effects are lost since there are no longer any rearrangements of the particles with respect to their nearest neighbors, while for strong disorder or the pinning dominated regime, the memory is also lost since the response is dominated by the pinning sites and not the vortex configurations. Our results should be general to other classes of particle-like systems interacting with quenched disorder and even for other systems that exhibit random organization or jammed phases.

## Data Availability

The data that support the plots within this paper and other findings of this study are available from the corresponding author upon request.
